# Detection of AXL expression in circulating tumor cells of lung cancer patients using an automated microcavity array system

**DOI:** 10.1002/cam4.2846

**Published:** 2020-01-30

**Authors:** Mio Ikeda, Yasuhiro Koh, Shunsuke Teraoka, Koichi Sato, Kuninobu Kanai, Atsushi Hayata, Nahomi Tokudome, Hiroaki Akamatsu, Yuichi Ozawa, Keiichiro Akamatsu, Katsuya Endo, Masayuki Higuchi, Masanori Nakanishi, Hiroki Ueda, Nobuyuki Yamamoto

**Affiliations:** ^1^ Internal Medicine III Wakayama Medical University Wakayama Japan; ^2^ Medical Business Sector Hitachi Chemical Co., Ltd. Chikusei Japan

**Keywords:** AXL, circulating tumor cells, epithelial‐to‐mesenchymal transition, liquid biopsy, lung cancer

## Abstract

Noninvasive diagnostics using circulating tumor cells (CTCs) are expected to be useful for decision making in precision cancer therapy. AXL, a receptor tyrosine kinase is associated with tumor progression, epithelial‐to‐mesenchymal transition (EMT), and drug resistance, and is a potential therapeutic target. However, the epithelial markers generally used for CTC detection may be not enough to detect AXL‐expressing CTCs due to EMT. Here, we evaluated the detection of AXL‐expressing CTCs using the mesenchymal marker vimentin with a microcavity array system. To evaluate the recovery of cancer cells, spike‐in experiments were performed using cell lines with varying cytokeratin (CK) or vimentin (VM) expression levels. With high CK and low VM‐expressing cell lines, PC‐9 and HCC827, the recovery rate of AXL‐expressing cancer cells was 1%‐17% using either CK or VM as markers. Whereas, with low CK and high VM‐expressing cell lines, MDA‐MB231 and H1299, it was 52%‐75% using CK and 72%‐88% using VM as a marker. For clinical evaluation, peripheral blood was collected from 20 non–small cell lung cancer patients and CTCs were detected using CK or VM as markers in parallel. Significantly more AXL‐expressing single CTCs were detected in VM‐positive than CK‐positive CTCs (*P* < .001). Furthermore, CTC clusters were identified only among VM‐positive CTCs in 20% of patients. Patients with one or more prior treatments harbored significantly more VM‐positive AXL‐expressing CTCs, suggesting the involvement of these CTCs in drug resistance. These results indicate the necessity of integrating mesenchymal markers with CTC detection and this should be further evaluated clinically.

## Graphical abstract

We established the detection of AXL‐expressing in circulating tumor cells of lung cancer patients incorporating vimentin as a CTC marker in addition to cytokeratin. Significantly more AXL‐expressing CTCs were detected in patients who had prior treatment history than those did not, suggesting the involvement of AXL in drug resistance.

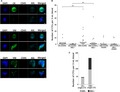

## 1 INTRODUCTION

Diagnosis and anticancer treatment monitoring of advanced solid tumors often involve tumor tissue biopsy, which is an invasive diagnostic procedure. Noninvasive diagnostic procedures are desired and diagnostics using circulating tumor cells (CTCs), which are tumor cells circulating in peripheral blood have been developed over the last decade.^1^, ^2^, ^3^ CTCs are thought to be metastatic cells from the solid tumor site and are one of the avenues for metastasis from the primary tumor site to distant tissues,^4^, ^5^ thus CTCs are associated with tumor progression and patient prognosis.^6^, ^7^ Moreover, because it is thought that CTCs reflect characteristics of the primary solid tumor such as genetic alterations and expression of therapeutic targets,^8^, ^9^ CTCs may be useful for estimating established biomarkers that can monitor response to treatment and the presence or lack of tumor‐specific therapeutic targets.^10^, ^11^, ^12^ Therefore, the detection and analysis of CTCs could be a minimally invasive diagnostic procedure for repetitive monitoring and deciding on therapeutic strategy. CellSearch is the only FDA‐approved CTC enumeration platform and has demonstrated the clinical utility of CTCs in a several cancers,^7^, ^13^, ^14^ however, their utility in non–small cell lung cancer (NSCLC) has been limited by the low expression of EpCAM in CTCs derived from NSCLC patients.^15^, ^16^ Lung cancer causes a large number of cancer‐related deaths world‐wide and is often diagnosed at an advanced stage.^17^

We previously reported on an automated microcavity array (MCA) system (Hitachi Chemical Co., Ltd.), which could efficiently detect CTCs in lung cancer patients with higher specificity compared to CellSearch.^18^ Because the MCA system can selectively isolate CTCs from peripheral blood based on the differences in size and deformability, the selection criteria can be tuned to match the properties of targeted proteins on CTCs.

AXL is receptor tyrosine kinase in the TAM (Tyro‐Axl‐Mer) family that is activated by the ligand growth arrest specific‐6 (Gas6). AXL is known to be an important factor driving epithelial‐to‐mesenchymal transition (EMT) and is involved in a wide variety of cellular responses including promoting cell proliferation, survival, adhesion, motility, and invasion.^19^, ^20^, ^21^ Moreover, the level of AXL expression is associated with cancer progression and drug resistance, thus new pharmaceuticals targeting AXL are being developed.^22^, ^23^, ^24^ The detection of AXL‐expressing CTCs in peripheral blood would be useful for deciding on therapeutic strategy. However, AXL is known to be the driving factor in EMT and many current technologies including CellSearch rely on epithelial‐specific markers, such as cytokeratin (CK), to detect CTCs, thus the down regulation of those markers due to EMT limits the detection of AXL‐expressing CTCs. Alternatively, mesenchymal‐specific markers may be useful for detecting these CTCs. In addition, because it is thought that CTCs expressing mesenchymal markers become more invasive and adhesive than those expressing only epithelial markers, there is a possibility that counts of mesenchymal marker positive CTCs may predict some clinical features and patient prognosis.

In this study, we established the detection of AXL‐expressing CTCs in advanced NSCLC patients using the mesenchymal‐specific marker vimentin (VM), with an MCA system. In these patients, VM‐positive AXL‐expressing CTCs are more frequently detected than CK‐positive AXL‐expressing CTCs.

## 2 MATERIALS AND METHODS

### 2.1 Cell lines and culture

The human NSCLC cell lines HCC827, NCI‐H1299, and the human breast cancer cell line MDA‐MB231 were purchased from the American Type Culture Collection (ATCC). The human NSCLC cell line PC‐9 was kindly provided by Dr Fumiaki Koizumi (Tokyo Metropolitan Komagome Hospital). The PC‐9, HCC827 and H1299 cell lines were cultured in RPMI‐1640 medium (Thermo Fisher Scientific) containing 10% fetal bovine serum (Sigma‐Aldrich) at 37°C with 5% CO_2_ supplementation. The MDA‐MB231 cell line was cultured in Dulbecco's modified Eagle medium (Thermo Fisher Scientific) containing 10% fetal bovine serum at 37°C with 5% CO_2_ supplementation.

### 2.2 Immunocytochemistry

Cells were seeded on an 8‐well chamber slide (Thermo Fisher Scientific) and left for more than 24 h to reach 50% confluence. Cells were fixed for 20 min with 4% paraformaldehyde (Sigma‐Aldrich) at room temperature and washed three times with PBS for 5 min each. The cells were then permeabilized for 5 min with 0.5% Triton X‐100 at room temperature. After washing with PBS, the cells were incubated with 2% BSA in PBS for 30 min for blocking and then incubated with a 1:500 dilution of vimentin V9 mouse mAb (sc‐6260, Santa Cruz Biotechnology) in 2% BSA for 1 h at room temperature. From here on, the cells were washed three times with PBS between each operation. The cells were incubated with Alexa Fluor 594‐labeled anti‐mouse secondary antibody from Hitachi chemical company for 30 min at room temperature. Next, the cells were incubated with 1:500 diluted AXL C89E7 rabbit mAb (#8661, Cell Signaling Technology) in 2% BSA for 1 h at room temperature, and then incubated with 1:500 diluted goat‐anti‐rabbit Alexa Fluor 647 (A‐21245, Thermo Fisher scientific) in 2% BSA for 30 min at room temperature. The cells were subsequently incubated with Alexa Fluor 488‐labeled anticytokeratin mouse mAb from Hitachi chemical company for 30 min at room temperature. Finally, the slide was mounted using VECTASHIELD with 4ʹ,6‐diamidino‐2‐phenylindole (DAPI) (H‐1200, Vector Laboratories) as a mounting media. Images were obtained using a fluorescence microscope, Axio Imager M2m and a digital camera, Axio Cam 503 mono (Carl Zeiss).

### 2.3 Western blotting

Cells were lysed in M‐PER Mammalian Protein Extraction Reagent (Thermo Fisher Scientific) supplemented with protease inhibitor cocktail (Thermo Fisher Scientific), 50 mM NaF and 2 mM Na_3_VO_4_. Protein concentration was evaluated with a BCA Protein Assay Kit (Thermo Fisher Scientific). Western blotting was performed using standard techniques; total cellular protein from the cells were loaded in each lane and separated on SDS‐PAGE. SDS‐PAGE gels were transferred onto PVDF membranes (Immobilon‐P, Millipore). The membranes were blocked with 5% milk in TBST and then incubated with primary antibody, 1:1000 diluted AXL C89E7 rabbit mAb (#8661), 1:500 diluted Vimentin V9 mouse mAb (sc‐6260), 1:500 diluted anti‐pan cytokeratin C11 mouse mAb (sc‐8018, Santa Cruz Biotechnology), or 1:4000 diluted anti‐β‐actin mouse mAb (sc‐47778, Santa Cruz Biotechnology), overnight at 4°C. The membranes were subsequently incubated with secondary antibodies, 1:2000 diluted goat‐anti‐rabbit HRP Conjugate (W401B, Promega) or 1:2000 diluted goat‐anti‐mouse HRP Conjugate (W402B, Promega) for 1 h at room temperature. Immunoblots were developed with Clarity Western ECL Substrate (Bio‐Rad) and visualized with a WSE‐6100 LuminoGraph (ATTO).

### 2.4 Cell spike‐in experiments

Cells were harvested with 0.25% trypsin/ethylenediaminetetraacetic acid (EDTA) (Thermo Fisher Scientific) at 37 °C before the blood spiking experiment. Zero, 40, or 200 cells were spiked into 6 mL of peripheral blood from healthy donors at Wakayama Medical University who had consented to donation in writing. Blood samples were divided into 3 mL per test and applied to an automated MCA system for cancer cells identification and AXL staining. In each experiment, cancer cells identification using anti‐CK or VM antibody as one of the CTC detection markers was performed in parallel dividing 3 mL spiked samples. All experiments were performed three times. To assess technical variance, three technical replicates containing 100 cells per 3 mL of blood were analyzed in parallel.

### 2.5 Tumor cell enrichment and detection

CTCs were enriched and immunostained using an automated MCA system.^18^, ^25^, ^26^ A whole blood sample was added to the reservoir of the MCA system. The blood sample was filtrated through the metal filter in the cartridge. The staining process is automated as follows: CD45 staining for 1 h, fixation for 10 min, permeabilization for 10 min, AXL staining for 90 min, and finally DAPI and CK or VM staining for 30 min. Four‐minute washes were carried out between each step and a 7‐min wash was done before the final step. Cell fixation, permeabilization, wash buffer and staining reagents for CD45, DAPI, and CK were provided by Hitachi chemical company. AXL was stained with the same antibody as immunocytochemistry and VM was stained with vimentin V9 Alexa Fluor 488 (sc‐6260 AF488, Santa Cruz Biotechnology). An image of the entire cell array area was captured using a fluorescence microscope (Axio Imager M2m; Carl Zeiss) with an integrated 10× objective lens and a computer‐operated motorized stage, a digital camera (AxioCam 503 mono; Carl Zeiss), and ZEN image acquisition software (Carl Zeiss). CTCs were defined as DAPI, CK or VM‐positive, and CD45‐negative cells.

### 2.6 Clinical study

Twenty patients with advanced NSCLC and 10 healthy donors were enrolled in this study and evaluated for CTC detection between May and December 2018 at Wakayama Medical University. The peripheral blood samples were collected in blood collection tubes (Becton Dickinson and Company) containing EDTA to prevent coagulation, and then processed by the MCA system within 3 h of blood draw. For each donor, the blood sample was divided into 3 mL portions used to run each CTC identification marker test, CK or VM, in parallel. This study was approved by the institutional review board at Wakayama Medical University and written informed consent was obtained from all donors. This study has been registered with the University Medical Hospital Information Network (UMIN) Clinical Trials Registry under the identifier UMIN000021712.

### 2.7 Statistical analysis

Statistical analyses were performed using GraphPad Prism 6 (GraphPad Software). A *P*‐value less than .05 was considered statistically significant.

## 3 RESULTS

### 3.1 Establishment of a method for detecting AXL‐expressing cancer cells using vimentin as a marker

**Figure 1:**
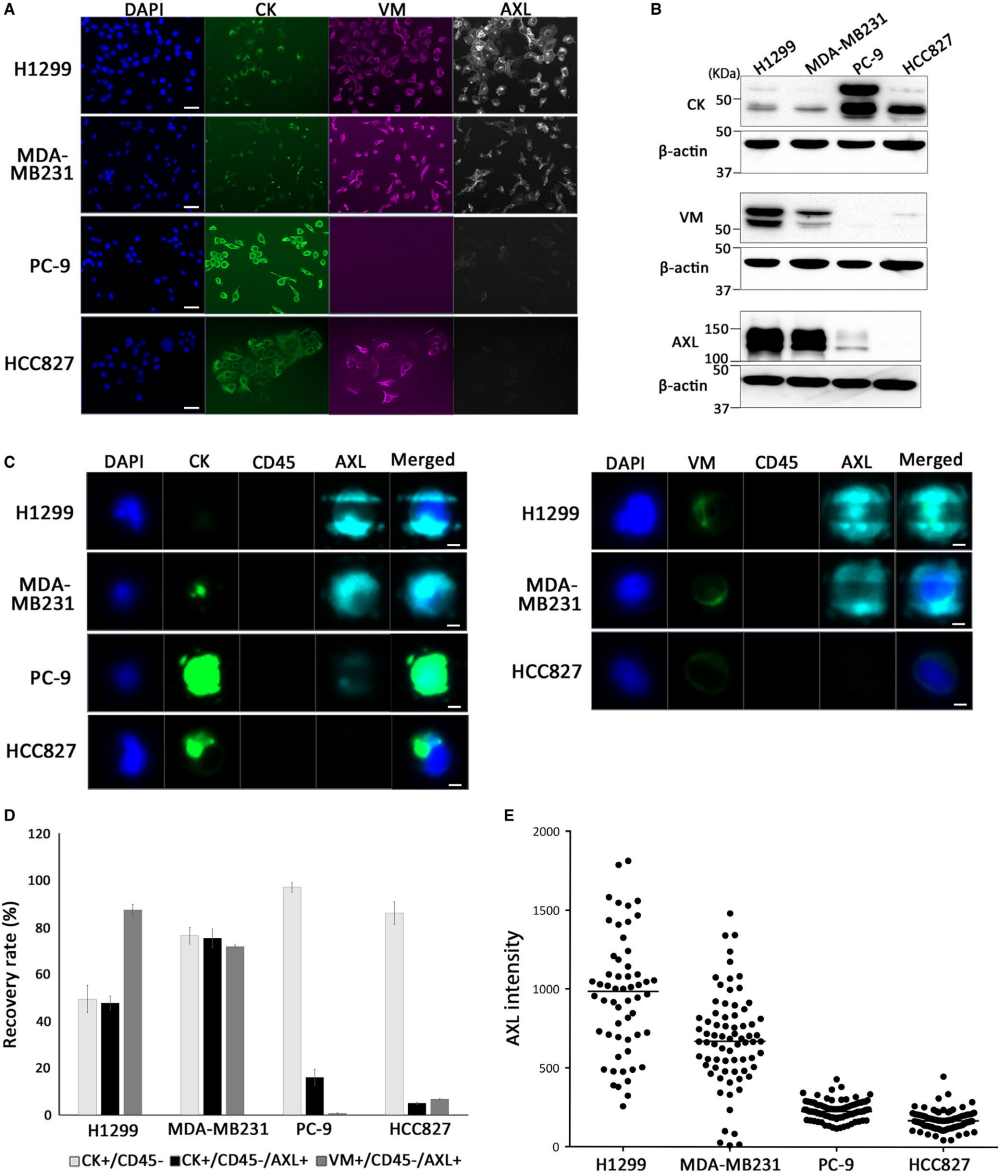
Establishment of a method for detecting AXL‐expressing cancer cells using spike‐in models. A, Immunostaining images of DAPI (blue), pan‐cytokeratin (green), vimentin (violet), and AXL (white) H1299, MDA‐MB231, PC‐9, and HCC827 cell lines cultured on a chamber slide. Scale bar represents 50 μm. B, Western blot analysis of pan‐cytokeratin, vimentin, and total AXL protein levels in four cancer cell lines. C, Representative immunostaining images of cancer cells detected using CK (left) or VM antibody (right). Scale bar represents 5 μm. D, Mean recovery rates of four spiked cancer cell lines from three replicates analyzed by the MCA system. E, Level of heterogeneous expression of AXL in each cell line

To establish the method for detecting AXL‐expressing cancer cells using preclinical models, we examined the expression levels of AXL, epithelial marker CK, and mesenchymal marker VM by immunofluorescent staining (Figure 1A) and immunoblotting (Figure 1B) in NSCLC cell lines PC‐9, HCC827, and H1299 and breast cancer cell line MDA‐MB231. Because the level of CK expression in MDA‐MB231 and H1299, which are high AXL‐expressing cell lines, is relatively low compared to PC‐9 and HCC827 cells, we also employed the mesenchymal‐specific marker, VM, in addition to CK which is a standard epithelial marker for detecting CTCs.

We examined the detection of CK or VIM‐positive AXL‐expressing cancer cells by the MCA system using spike‐in models. Figure 1C shows representative images of immunofluorescence staining of each cell line recovered on the filter of cartridge of the MCA system. DAPI, CK, or VIM‐positive and CD45‐negative cells were defined as cancer cells. Among images of VM‐positive cancer cells (Figure 1C, right), very few PC‐9 cells were recovered because of their lack of VM expression. The mean recovery rate of three replicates of each cell line is shown in Figure 1D. When CK was used as a marker, AXL expression was detected in 5% and 17% of the high CK‐expressing cells, HCC827 and PC‐9, respectively, and was detected in 47% and 75% of the low CK‐expressing cells, H1299 and MDA‐MB231, respectively. Whereas, when VM was used as a marker, AXL expression was detected in 72% and 88% of VM‐expressing MDA‐MB231 and H1299 cells, respectively, but only detected in 1% and 7% of PC‐9 and HCC827 cells with low VM expression, respectively. This result suggests that expression of VM is significantly higher in AXL‐expressing cells compared to the expression level of CK, and CTCs may be detectable in cancer patients using VM expression. Nevertheless, the correlation between the fluorescence intensity of AXL and CK or VM was very weak (correlation coefficient was *r* = −.30 for CK and *r* = −.23 for VM) (Figure S1). Figure 1E shows the fluorescence intensity of AXL expression in each cancer cell line. The expression level of each cell is obviously different even within the AXL‐expressing monoclonal cell lines H1299 and MDA‐MB231.

To test the robustness of the system, the linearity of detection rate was evaluated using H1299 and HCC827 cells, and good correlation was observed between the recovered number and the expected number with a correlation coefficient (*R* ^2^) of 0.99 (Figure S2A). Very little technical variance was confirmed with the results from three technical replicates using H1299 or HCC827cells (Figure S2B). We also confirmed that physical pressure caused by MCA system had little effect on AXL expression by comparing the AXL‐positivity rates of cultured cells and detected cells by MCA system (Figure S2C).

### 3.2 Enumeration of AXL‐expressing CTCs in lung cancer patients

A total of 20 advanced NSCLC patients and 10 healthy volunteers were enrolled in this study at Wakayama Medical University Hospital. The demographics of the patients are shown in Table 1 were as follows: median age was 70 years (range, 54‐82); 80% male; stage III/IV, 10/90%; Adenocarcinoma/Squamous cell carcinoma/other, 65/30/5%. The details of each patient are shown in Table S1.

**Table 1:** Patient characteristics (n = 20)

Number of patients	20
Age: median, (range) years	70 (54‐82)
Gender: n, (%)	
Male	16 (80)
Female	4 (20)
Histological type: n, (%)	
Adenocarcinoma	13 (65)
‐ EGFR mutated	6 (30)
Squamous cell carcinoma	6 (30)
Other	1 (5)
Stage: n, (%)	
III	2 (10)
IV	18 (90)
Performance status: n, (%)	
0	2 (10)
1	15 (75)
≥2	3 (15)
Previous therapies: n, (%)	
0	13 (65)
1	3 (15)
≥2	4 (20)

The patients were evaluated for CTCs, nine patients were evaluated before their first therapeutic treatment, one patient was evaluated in treatment, and 10 patients were evaluated after progressive disease (PD) status was determined for the last treatment. The peripheral blood sample from each patient was divided and analyzed by staining with CK or VM antibody in parallel for every analysis. CTCs were enriched and AXL expression in CK or VM‐positive CTCs was quantified using the MCA system.

**Figure 2:**
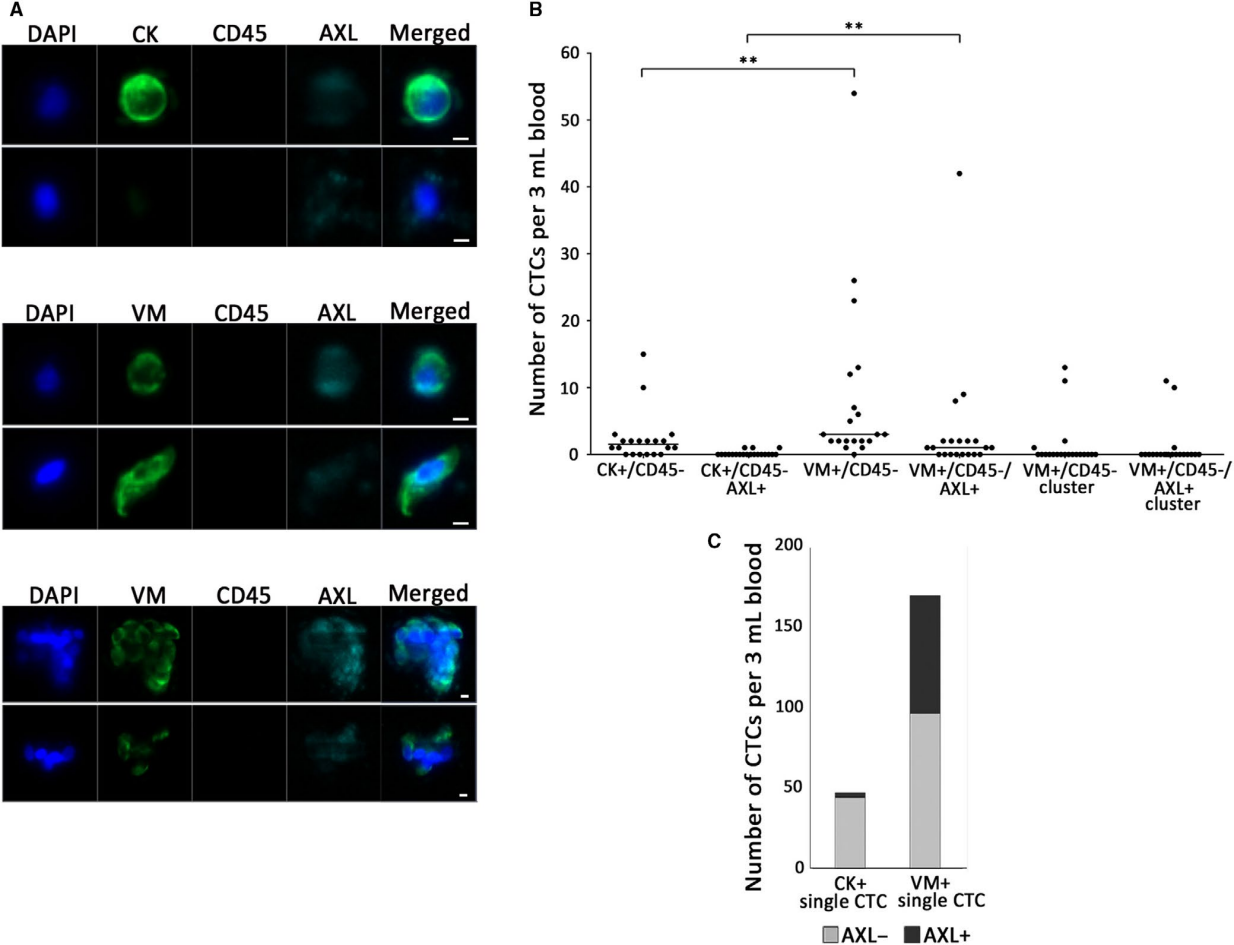
Number of CTCs and detection of AXL expression on CTCs in patient samples (n = 20). A, Representative images of CK‐positive (upper) or VM‐positive (middle) AXL‐expressing single CTCs or VM‐positive AXL‐expressing CTC clusters (lower) from patient samples. Scale bar represents 5 μm. B, Distribution of each CTC type in all cancer patient samples. The number of CK‐positive single CTCs include AXL‐expressing CK‐positive single CTCs as do VM‐positive single CTCs and CTC clusters. Single CTCs and CTC clusters were counted separately. Each blood sample was divided for analysis of CK or VM antibody. VM‐positive single CTCs and CTC clusters were detected from the same analysis. **P < .01. C, The total number of detected single CTCs. There is significant difference between the ratio of AXL‐expressing VM‐positive CTCs to VM‐positive CTCs and the ratio of AXL‐expressing CK‐positive CTCs to CK‐positive CTCs. *P* < .001

Figure 2A shows representative images of single CTCs classified as CK‐positive or VM‐positive (top and middle) and VM‐positive CTC clusters (bottom). The distribution of each CTC type is shown in Figure 2B and the breakdown of the CTC counts in each patient is shown in Table S1. CK‐positive single CTCs were detected in 70% of patients (median, 1.5; range, 0‐15). AXL‐expressing CK‐positive single CTCs were detected in 15% of patients (median, 0; range, 0‐1). On the other hand, VM‐positive single CTCs were detected in 95% of patients (median, 3; range, 0‐54) and AXL‐expressing VM‐positive single CTCs were detected in 60% of patients (median, 1; range, 0‐42). In the 10 healthy donors, although few CK‐positive cells were detected (median, 0; range, 0‐1), slightly more VM‐positive cells were detected (median, 0.5; range, 0‐4), suggesting the possibility of VM positivity on nontumor cells. Nonetheless, AXL‐expressing CK‐positive CTCs were not detected in the healthy donors and AXL‐expressing VM‐positive CTCs were detected in only 2 individuals with a cell count of 1 cell in 3 mL of blood (Figure S3).

There is significant difference between the number of CK‐positive CTCs and VM‐positive CTCs regardless of presence or absence of AXL expression (*P* < .01) and the ratio of AXL‐expressing single CTCs was also significantly higher in VM‐positive CTCs than in CK‐positive CTCs (*P* < .001) (Figure 2B,C). Notably, CTC clusters were identified in 20% of patients that were positive for VM but not CK (median, 0; range, 0‐11). VM‐positive CTC clusters with AXL expression were detected in 15% of patients (median, 0; range, 0‐11) (Figure 2B).

**Figure 3:**
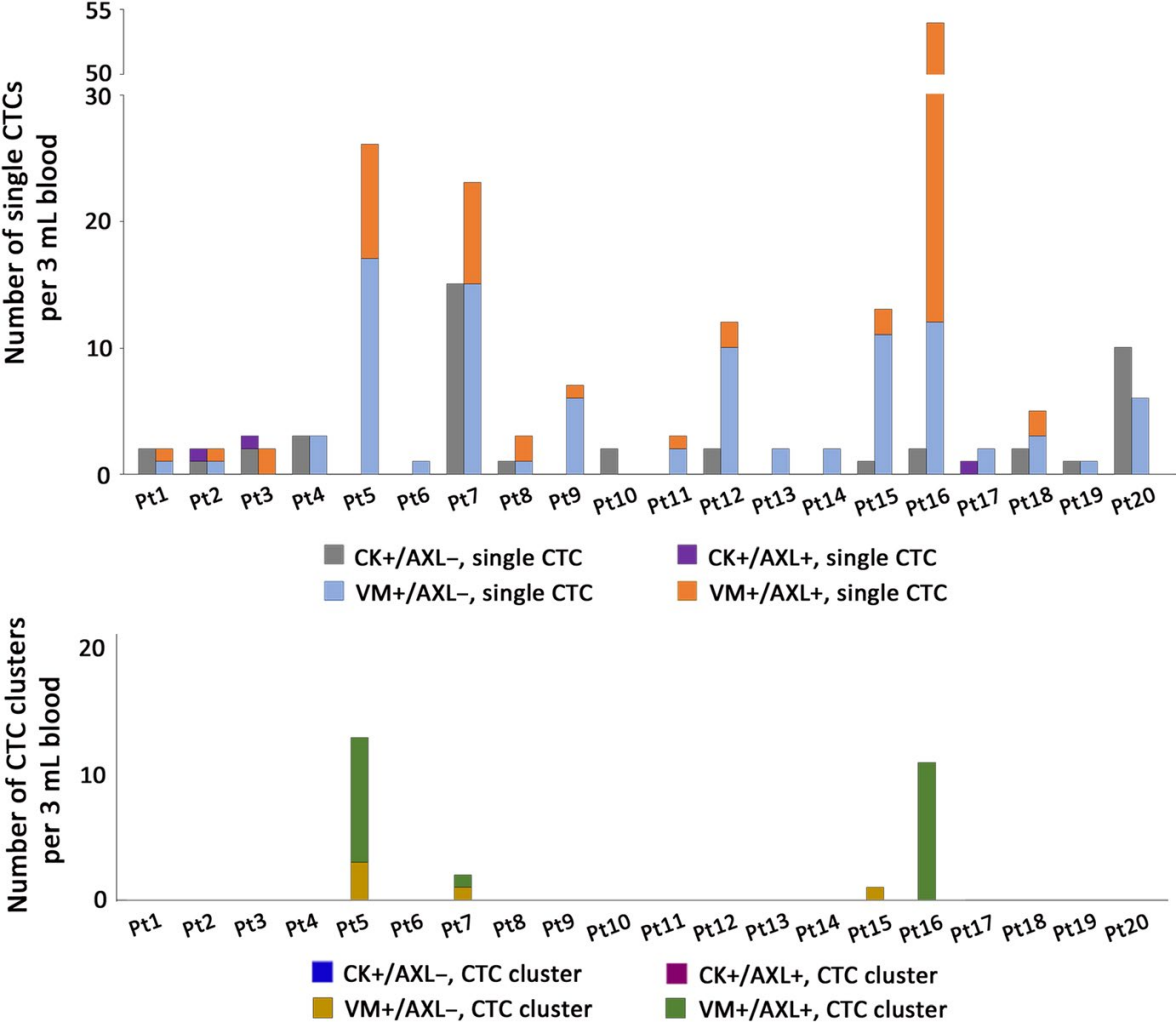
Number of AXL‐positive and ‐negative single CTCs (upper) and CTC clusters (lower) in each patient sample. Each bar shows the number of CTCs with AXL‐negative CK‐positive single CTCs in gray, AXL‐positive CK‐positive single CTCs in violet, AXL‐negative VM‐positive single CTCs in blue, AXL‐positive VM‐positive single CTCs in orange, AXL‐negative VM‐positive CTC clusters in brown, and AXL‐positive VM‐positive CTC clusters in green

The proportion of AXL‐positive and ‐negative CTCs in each patient for single CTCs (upper) or CTC clusters (lower) is illustrated in Figure 3. Although the blood sample for staining with CK or VM antibodies was concurrently processed in parallel for each donor, the total number of VM‐positive CTCs was more than twice the total number of CK‐positive CTCs in 12 patients.

### 3.3 Clinicopathologic correlation

**Figure 4:**
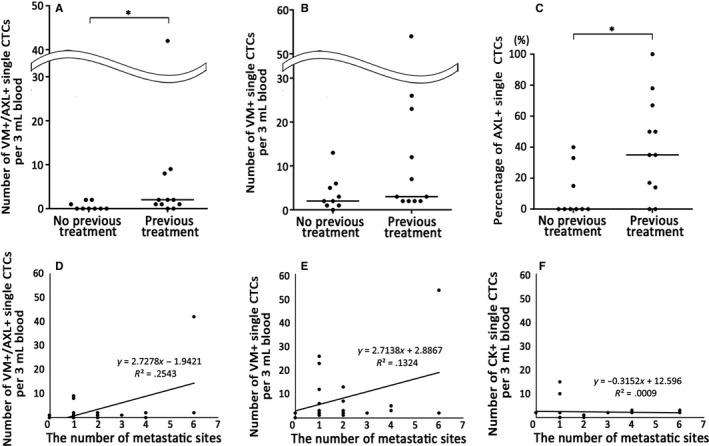
Clinicopathologic correlation with CTCs detected in patients. A, Comparison of the number of AXL‐expressing VM‐positive single CTCs between patients with (n = 11) and without previous therapeutic treatments (n = 9); *P < .05. B, Comparison of the number of VM‐positive single CTCs regardless of AXL expression between patients with and without previous therapeutic treatments; *P* = .12. C, Comparison of the ratio of AXL‐expressing VM‐positive single CTCs to VM‐positive single CTCs in each patient between patients with and without previous therapeutic treatments; *P < .05. Correlation between the number of distant metastatic sites and the number of AXL‐expressing VM‐positive single CTCs (*r* = .50, *P* < .05) (D), VM‐positive single CTCs (*r* = .36, *P* = .11) (E), and CK‐positive single CTCs (*r* = −.044, *P* = .85) (F)

Clinicopathologic analyses were performed on several factors correlating with the number of CTCs. Interestingly, significantly more AXL‐expressing VM‐positive single CTCs were detected in patients with (n = 11, median = 2) than without previous therapeutic treatment (n = 9, median = 0) (*P* < .05) (Figure 4A), whereas therapeutic history made no significant on the number of VM‐positive single CTCs (Figure 4B). No correlation with therapeutic history was observed for CK‐positive single CTCs (Figure S4). Moreover, AXL‐positivity rates in single VM‐positive CTCs between patients with previous treatment and those without were compared and there was also a statistically significant difference (*P* < .05) (Figure 4C).

Figure 4D‐F show the correlation between the number of single CTCs and the number of distant metastatic sites in all patients. A distant metastatic site was defined as a metastatic site determined to be at stage IV for metastasis. There was a correlation between the number of AXL‐expressing VM‐positive single CTCs and distant metastatic sites (correlation coefficient was *r* = .50, *P* < .05) (Figure 4D). For VM‐positive single CTCs, there was weakly correlation between CTC counts and the number of metastatic sites (correlation coefficient was *r* = .36, *P* = .11) (Figure 4E). Among CK‐positive single CTCs, no correlation was observed between CTC counts and the number of distant metastatic sites (*r* = −.044, *P* = .85) (Figure 4F).

We also assessed the impact of AXL‐expressing CTCs on the following treatment in 17 patients from whom we obtained the response data (Table S1). Thirteen patients had partial response (PR) or stable disease (SD) and 3 had progressive disease (PD). Cut‐off value for segregating PR/SD and PD was 45% of AXL‐positivity on CTCs according to receiver operating characteristic curve (Figure S5). With this cut‐off, though there was a trend that patients with more AXL‐positive CTCs were likely to have PD, it was not statistically significant (*P* = .071).

## 4 DISCUSSION

In this study, we successfully detected the expression of AXL on CTCs and compared CTCs identified by epithelial‐specific marker CK and mesenchymal‐specific marker VM for differences in the number and degree of AXL‐positive cells. We demonstrated that significantly more AXL‐expressing CTCs were detected among VM‐positive CTCs than CK‐positive CTCs, indicating that incorporating mesenchymal markers is required for better detection of AXL‐expressing CTCs using an automated MCA system.

Repetitive acquisition of tumor specimens for monitoring is known to be difficult. Therefore, diagnosis and prognosis using CTCs in peripheral blood, a so‐called liquid biopsy, is needed as an easily and minimal invasive clinical procedure. For liquid biopsies, circulating tumor‐derived DNA (ctDNA) is also an important actor which is currently approved for epidermal growth factor receptor (EGFR) mutation testing and is useful for genomic analyses.^27^ Alternatively, CTCs have the advantage over ctDNA of being able to measure their protein expression, which can become a target of cancer therapies.^3^ It is reported that the expression of programmed death 1 (PD‐1) can be detected on CTCs and potentially used to predict for efficacy.^28^, ^29^, ^30^

AXL expression in tumor tissues has been reported to correlate with tumor progression, poor prognosis, and drug resistance in various cancer and drug settings.^21^, ^31^, ^32^, ^33^, ^34^, ^35^ Therefore, AXL expression level has a potential to be utilized as a useful biomarker for patient survival and monitoring emerging resistance to treatment. Moreover, AXL‐targeting agents have been developed to overcome drug resistance and their clinical evaluation is ongoing. We previously reported that an automated MCA system with CK staining can efficiently detect CTCs in lung cancer patients compared to the CellSearch system.^18^ However, AXL‐expressing CTCs may undergo EMT that cause down regulation of epithelial‐specific marker expression. Therefore, we employed VM as a marker in the present work. The results of this study support the hypothesis that AXL‐expressing CTCs may have induced EMT and that it is difficult to detect these cells using epithelial‐specific markers due to the significant reduction in expression. In addition, there was more than twice the number of VM‐positive CTCs as CK‐positive CTCs in 12 patient samples (Figure 3). Moreover, the CTC clusters detected in 20% of the patients were only VM‐positive CTCs and the few DAPI‐positive, CK‐negative, CD45‐negative, and AXL‐expressing cell clusters were only present in some cases (data not shown). This is consistent with the previous report that CTC clusters have a higher metastatic potential than single CTCs.^36^, ^37^ Many current technologies including CellSearch rely on epithelial‐specific markers to detect CTCs; however, it seems that the integration of mesenchymal‐specific markers like VM with CTC detection is necessary to precisely detect CTCs in peripheral blood.

In order to evaluate the significance of AXL‐expressing CTCs that may have undergone EMT, detection of such CTCs needs to be optimized. Methods for isolating mesenchymal‐like CTCs have been developed including immunobinding methods.^38^, ^39^, ^40^ CTCs are, however, highly heterogeneous, thus CTCs that can be isolated by immunobinding methods utilizing EpCAM targeting in particular may be limited to the subgroup expressing the marker of interest at a considerable level. On the other hand, filter‐based methods including our MCA system are not surface marker dependent and used for isolation of mesenchymal‐like CTCs as well.^41^, ^42^, ^43^, ^44^ It is also important to select appropriate mesenchymal markers to detect mesenchymal‐like CTCs after capturing them. Among various EMT markers, VM is thought to be one of the general and well‐studied mesenchymal markers and is used in many studies.^45^, ^46^ Other potential markers such as CD133, one of the cancer stem cell markers have been employed for mesenchymal‐like CTCs isolation methods.^40^ There are many reports indicating that CTCs expressing high levels of EMT markers in several cancer types such as breast, prostate, and colorectal, are often accompanied by the expression of cancer stem cell markers, suggesting the utility of incorporating stem cell markers such as CD133.^47^, ^48^, ^49^ However, the relationship between EMT markers and CD133 on CTCs in lung cancer is not well‐understood. Potential of other EMT markers including CD133 to detect mesenchymal‐like CTCs should be pursued in the future, nonetheless.

There are several filter‐based systems for capturing CTCs.^50^, ^51^, ^52^ We did not directly compare the performance between the MCA system and other filter‐based methods but preclinical and clinical data support that the MCA system is comparable to other systems and it may have an advantage over others in NSCLC. It was previously shown that in spike‐in experiments MCA system could recover more than 80% of various lung cancer cell lines with the mean size of 12.5 μm and more including high VM‐expressing cell lines, such as PC‐14.^25^ Moreover, 88% of spiked H1299 cells was recovered in this study (Figure 1D). Therefore, we concluded that MCA system can efficiently capture and detect both epithelial and mesenchymal tumor cells. However, results from our previous studies using MCA system showed that the MCA system tended to miss smaller cells such as small cell lung cancer.^18^, ^26^ Krebs et al also reported that filtration‐based CTC detection was size‐dependent and it may miss cells less than 8 μm in size.^53^ Physical properties of tumor cells such as size and stiffness play a pivotal role in capture efficiency and it should be taken into consideration for further device development.

AXL expression on CTCs has previously been detected in peripheral blood from prostate tumor patients using RT‐PCR on single CTCs isolated using a micromanipulator device from CTCs enriched by microfiltration.^54^ In that study, AXL expression was analyzed as one of the EMT‐related and drug targets for evaluating heterogeneous expression profiles among single CTCs.

It is known that tumor heterogeneity is frequent in cancer.^55^, ^56^ In this study, AXL‐expressing VIM‐positive CTCs were detected in 60% of patient samples and all except one of them also contained AXL‐negative VM‐positive CTCs, suggesting that there is heterogeneity of AXL expression on CTCs in a single patient. Even monoclonal cell lines showed heterogeneous AXL expression in this study (Figure 1E). Moreover, the expression levels of CK and VM in H1299 cells also covered a wide range; however, there was little correlation between the expression level of those markers and AXL (Figure S1). Since AXL is involved in many cellular responses, the expression level of AXL may be easily affected by external stimuli. These observations indicate that AXL expression needs to be further studied.

As mentioned above, it was reported that AXL expression is involved in drug resistance in many settings including EGFR tyrosine kinase inhibitors, which are used to treat tumors in advanced NSCLC patients harboring the EGFR mutation^33^, ^35^ and traditional therapies, such as chemotherapy and radiotherapy.^57^, ^58^, ^59^ In this study, though the enrolled patients have varying clinical backgrounds and had received various kinds of therapeutic treatments (Table S1), both the number and percentage of AXL‐expressing VM‐positive CTCs but not the number of VM‐positive CTCs were significantly different in patients with one or more prior treatment and those without a history of treatment (Figure 4A,C). Moreover, AXL‐expressing CTC clusters were detected in specimens with more than two forms of therapeutic treatment. These data suggest that cancer shifts to a mesenchymal phenotype and AXL‐expressing CTCs and CTC clusters increase during the process of acquiring various drug resistances depending on the increase in the number of therapies. It was also observed that the number of VM‐positive single CTCs but not the number of CK‐positive single CTCs was correlated with the number of distant metastatic sites in patients (Figure 4B). This result indicates that CTC detection using a mesenchymal‐specific marker rather than an epithelial‐specific marker has greater clinical utility. In addition, significantly more metastatic sites were identified in patients harboring AXL‐expressing VM‐positive single CTCs compared to those harboring VM‐positive single CTCs, supporting previous reports that AXL accelerates metastasis.

There are a few limitations in this study. First, the sample size of the clinical study is small and the background of collected blood samples were highly nonuniformly. Therefore, the findings should be validated in another cohort using more unified samples. Second, it is inevitable that VM staining tends to increase false positives more than conventional CK staining even when CD45 staining is used to exclude leukocytes because VM is also expressed on leukocytes. However, as AXL‐expressing VM‐positive cells were only detected in 2 donors with 1 cell per sample, we think this method is at least reliable for detecting AXL expression on CTCs. Nevertheless, further evaluation in a larger patient cohort is warranted. Third, it remains unclear whether VM and AXL expression on CTCs correlates to those in primary tumor tissue. Paired sample collection of tissue and blood is necessary to address this question. Fourth, with regard to the involvement of increased AXL expression on CTCs in drug resistance phenotype, collection of blood samples before the treatment and after disease progression is necessary.

In summary, we established a method for detecting AXL‐expressing CTCs with an MCA system and the number of AXL‐expressing VM‐positive CTCs significantly correlated with the number of distant metastatic sites. Detection of AXL‐expressing CTCs may also have a potential to be indicative of poor prognosis and acquired drug resistance, though further investigation is warranted. This is the first report of a method to detect AXL expression on CTCs in patients with lung cancer combined with VM expression as a marker. Our data suggest that integration of a mesenchymal marker like VM into CTC detection methods is necessary for precise detection. This would promote the development and clinical utility of liquid biopsies using CTCs.

## DISCLOSURE

Yasuhiro Koh received research funding from Daiichi Sankyo (TaNeDS). Masayuki Higuchi and Katsuya Endo are employees of Hitachi Chemical Co., Ltd.

## Data Availability

The data that support the findings of this study are available on request from the corresponding author. The data are not publicly available due to privacy or ethical restrictions.
