# Supplementary material for: Detection of AXL expression in circulating tumor cells of lung cancer patients using an automated microcavity array system
Source: Cancer Med. 2020 Jan 30;9(6):2122–33. doi: 10.1002/cam4.2846 (PMC7064033; doi:10.1002/cam4.2846)
Supplement: Supplementary file 6 [file CAM4-9-2122-s006.docx]

Table S1. Clinicopathological findings.

| Pt# | Gender | Age | Histological type | Stage | Previous therapies | | | | Number of CTC / 3 mL blood | | | Best response of  next therapy  after CTC detection | Distinct metastatic sites |
| --- | --- | --- | --- | --- | --- | --- | --- | --- | --- | --- | --- | --- | --- |
|  |  |  |  |  |  |  |  |  | CK+ single CTC | VM+ single CTC | VM+ CTC cluster |  |  |
|  |  |  |  |  | First line | Second line | Third line | Forth line | AXL+  of the above | AXL+  of the above | AXL+  of the above |  |  |
| 1 | M | 80 | Squamous cell carcinoma | III | Pembrolizumab |  |  |  | 2 | 2 | 0 | PR | Noね |
|  |  |  |  |  |  |  |  |  | 0 | 1 | 0 |  |  |
| 2 | M | 70 | Squamous cell carcinoma | IV | CDDP+GEM |  |  |  | 2 | 2 | 0 | PD | Pleural effusion, Cardiac effusion, Chest wall |
|  |  |  |  |  |  |  |  |  | 1 | 1 | 0 |  |  |
| 3 | F | 75 | Squamous cell carcinoma | IV | CBDCA+nab-PTX | Atezolizumab |  |  | 3 | 2 | 0 | Decease without next therapy | Abdominal nodules, Chest wall subcutaneous nodules, |
|  |  |  |  |  |  |  |  |  | 1 | 2 | 0 |  | Arm muscle, Gluteus maximus muscle, Kidney, Pleural |
| 4 | M | 72 | Adenocarcinoma | IV | CDDP+VNR | Atezolizumab |  |  | 3 | 3 | 0 | Transferred without  Next therapy | Pleural effusion, Adrenal gland, Brain |
|  |  |  |  |  |  |  |  |  | 0 | 0 | 0 |  | Mesenteric lymph node |
| 5 | M | 67 | Adenocarcinoma | IV | CDDP+VNR | weekly nab-PTX | Atezolizumab |  | 0 | 26 | 13 | SD | Contralateral lung |
|  |  |  |  |  |  |  |  |  | 0 | 9 | 10 |  |  |
| 6 | M | 62 | Adenocarcinoma | IV |  |  |  |  | 0 | 1 | 0 | PR | Parasternal lymph node |
|  |  |  |  |  |  |  |  |  | 0 | 0 | 0 |  |  |
| 7 | M | 75 | Squamous cell carcinoma | IV | CBDAC+nab-PTX | Atezolizumab |  |  | 15 | 23 | 2 | Decease without next therapy | Liver |
|  |  |  |  |  |  |  |  |  | 0 | 8 | 1 |  |  |
| 8 | M | 71 | LCNEC | IV | CDDP+ETP 4c+RT | CDDP+ETP | AMR | weekly CPT-11 | 1 | 3 | 0 | PR | Liver, Hepatic portal region lymph node |
|  |  |  |  |  |  |  |  |  | 0 | 2 | 0 |  |  |
| 9 | F | 72 | Adenocarcinoma | IV | CDDP+PEM |  |  |  | 0 | 7 | 0 | SD | upper lobe of contralateral lung, Adrenal gland |
|  |  |  |  |  |  |  |  |  | 0 | 1 | 0 |  |  |
| 10 | M | 68 | Squamous cell carcinoma | III |  |  |  |  | 2 | 0 | 0 | PR | None |
|  |  |  |  |  |  |  |  |  | 0 | 0 | 0 |  |  |
| 11 | M | 73 | Adenocarcinoma | IV |  |  |  |  | 0 | 3 | 0 | PR | Brain |
|  |  |  |  |  |  |  |  |  | 0 | 1 | 0 |  |  |
| 12 | M | 68 | Adenocarcinoma | IV | CDDP+PEM | DTX | Erlotinib+BEV |  | 2 | 12 | 0 | SD | Bone |
|  |  |  | EGFR L747-A750del+insP | |  |  |  |  | 0 | 2 | 0 |  |  |
| 13 | M | 54 | Adenocarcinoma | IV |  |  |  |  | 0 | 2 | 0 | PR | Pleural |
|  |  |  | EGFR L858R |  |  |  |  |  | 0 | 0 | 0 |  |  |
| 14 | F | 82 | Adenocarcinoma | IV | Erlotinib+BEV |  |  |  | 0 | 2 | 0 | PR | Pleural |
|  |  |  | EGFR Deletion 19 |  |  |  |  |  | 0 | 0 | 0 |  |  |
| 15 | M | 67 | Adenocarcinoma | IV |  |  |  |  | 1 | 13 | 1 | PR | Intraperitoneal lymph nodes, Adrenal gland |
|  |  |  |  |  |  |  |  |  | 0 | 2 | 0 |  |  |
| 16 | M | 69 | Adenocarcinoma | IV | Gefinitib | Zpi+Nivo | CBDCA+nab-PTX |  | 2 | 54 | 11 | PD | Pleural effusion, Cardiac effusion, Brain, Bone, |
|  |  |  | EGFR Deletion 19 |  |  |  |  |  | 0 | 42 | 11 |  | Intraperitoneal lymph nodes, Para-aortic lymph node |
| 17 | M | 78 | Adenocarcinoma | IV |  |  |  |  | 1 | 2 | 0 | PD | Liver, Bone |
|  |  |  |  |  |  |  |  |  | 1 | 0 | 0 |  |  |
| 18 | M | 67 | Adenocarcinoma | IV |  |  |  |  | 2 | 5 | 0 | PR | Pleural，Contralateral lung, Adrenal gland, Bone |
|  |  |  | EGFR Deletion 19 |  |  |  |  |  | 0 | 2 | 0 |  |  |
| 19 | M | 70 | Squamous cell carcinoma | IV |  |  |  |  | 1 | 1 | 0 | PR | Bone, Adrenal gland |
|  |  |  |  |  |  |  |  |  | 0 | 0 | 0 |  |  |
| 20 | F | 66 | Adenocarcinoma | IV |  |  |  |  | 10 | 6 | 0 | SD | Bone |
|  |  |  | EGFR L858R |  |  |  |  |  | 0 | 0 | 0 |  |  |

The number of CK-positive CTCs includes the number of AXL-expressing CK-positive CTCs as do VM-positive single CTCs and CTC clusters. Single CTCs and CTC clusters were counted separately.
